# The Identification of CELSR3 and Other Potential Cell Surface Targets in Neuroendocrine Prostate Cancer

**DOI:** 10.1158/2767-9764.CRC-22-0491

**Published:** 2023-08-03

**Authors:** Lucie Van Emmenis, Sheng-Yu Ku, Kaitlyn Gayvert, Jonathan R. Branch, Nicholas J. Brady, Subhasree Basu, Michael Russell, Joanna Cyrta, Aram Vosoughi, Verena Sailer, Hussein Alnajar, Etienne Dardenne, Elena Koumis, Loredana Puca, Brian D. Robinson, Michael D. Feldkamp, Annmarie Winkis, Nathan Majewski, Brent Rupnow, Marco M. Gottardis, Olivier Elemento, Mark A. Rubin, Himisha Beltran, David S. Rickman

**Affiliations:** 1 Department of Pathology and Laboratory Medicine, Weill Cornell Medicine, New York, New York.; 2 Department of Medical Oncology, Dana-Farber Cancer Institute, Boston, Massachusetts.; 3 Department of Physiology and Biophysics, Weill Cornell Medical College, New York, New York.; 4 Caryl and Israel Englander Institute for Precision Medicine, New York-Presbyterian Hospital, New York, New York.; 5 Janssen Research & Development, Spring House, Pennsylvania.; 6 Department for BioMedical Research, University of Bern, Bern, Switzerland.; 7 Department of Pathology, H. Lee Moffitt Cancer Center and Research Institute, Tampa, Florida.; 8 Meyer Cancer Center, Weill Cornell Medicine, New York, New York.; 9 Bern Center for Precision Medicine, University of Bern, Bern, Switzerland.

## Abstract

Although recent efforts have led to the development of highly effective androgen receptor (AR)-directed therapies for the treatment of advanced prostate cancer, a significant subset of patients will progress with resistant disease including AR-negative tumors that display neuroendocrine features [neuroendocrine prostate cancer (NEPC)]. On the basis of RNA sequencing (RNA-seq) data from a clinical cohort of tissue from benign prostate, locally advanced prostate cancer, metastatic castration-resistant prostate cancer and NEPC, we developed a multi-step bioinformatics pipeline to identify NEPC-specific, overexpressed gene transcripts that encode cell surface proteins. This included the identification of known NEPC surface protein CEACAM5 as well as other potentially targetable proteins (e.g., HMMR and CESLR3). We further showed that cadherin EGF LAG seven-pass G-type receptor 3 (CELSR3) knockdown results in reduced NEPC tumor cell proliferation and migration *in vitro*. We provide *in vivo* data including laser capture microdissection followed by RNA-seq data supporting a causal role of CELSR3 in the development and/or maintenance of the phenotype associated with NEPC. Finally, we provide initial data that suggests CELSR3 is a target for T-cell redirection therapeutics. Further work is now needed to fully evaluate the utility of targeting CELSR3 with T-cell redirection or other similar therapeutics as a potential new strategy for patients with NEPC.

**Significance:**

The development of effective treatment for patients with NEPC remains an unmet clinical need. We have identified specific surface proteins, including CELSR3, that may serve as novel biomarkers or therapeutic targets for NEPC.

## Introduction

Emerging data from metastatic biopsies of patients with metastatic castration-resistant prostate cancer (CRPC) suggest that up to 15% of patients develop neuroendocrine prostate cancer (NEPC) in later stages of prostate cancer, and this is higher after potent androgen receptor (AR)-targeting therapies (1–5). NEPC tumors are histologically heterogeneous but can appear morphologically similar to other poorly differentiated neuroendocrine carcinomas such as small cell lung cancer (SCLC) and typically expresses classical neuroendocrine markers such as chromogranin A/B (CHGA/CHGB), CD56 (NCAM1; refs. 6, 7), INSM1, and/or synaptophysin (SYP; ref. 8). Collective data support NEPC arising as a mechanism of resistance to AR therapies with loss of AR protein expression and/or canonical AR signaling in NEPC tumors and acquisition of alternative lineage programs that drive tumor growth (5, 8–12). During the transition from prostate adenocarcinoma to NEPC, mixed features can be seen with both adenocarcinoma and small cell carcinoma elements present on tumor biopsy; AR expression may also be retained in some cells but is generally lost; and rapid progression is typically seen, often to visceral organs. Patients are often treated using SCLC-based chemotherapy regimens, but responses are limited and prognosis is poor (5, 13, 14). Despite sharing histologic and molecular features with SCLC (such as *TP53* and *RB1* loss), NEPC retains many of the early genomic alterations that arise in prostate cancer [e.g., *TMPRSS2-ERG* gene rearrangement (4, 9, 11, 15–19)]. Recently, other distinct molecular subgroups of treatment-resistant tumors have been described which are either composed of tumor cells coexpressing both AR and neuroendocrine markers (amphicrine prostate cancer) or those that are double-negative for both (3, 20). As a newer generation of inhibitors are developed to overcome on-target resistance, it may be that the prevalence of NEPC will rise precipitously.

Though prostate cancer is one exemplar cancer, the tendency of cancers to develop lineage plasticity may be much more widespread and even occur outside of the targeted therapy context. The identification of lineage-associated, cancer subgroup-specific surface proteins will enable the development of more refined therapeutic options such as antibody-mediated therapies. This could include antibody–drug conjugates (ADC), chimeric antigen receptor T cells (CAR-T) and bispecific antibodies. Theranostics are widely used for targeting prostate-specific membrane antigen (PSMA) for the detection and treatment of advanced prostate adenocarcinoma (21–26). Cell surface targets enriched in NEPC include delta-like ligand 3 (DLL3; ref. 27) and CEACAM5 (28) which has led to new therapeutic approaches now being tested in clinical trials. However, the full spectrum of cell surface proteins is underexplored in NEPC. Herein, we address this unmet need by interrogating RNA-sequencing (RNA-seq) data from a large patient cohort of well-characterized prostate cancer samples including localized prostate adenocarcinoma, metastatic CRPC, and NEPC tumors (9). A computational framework was developed to include publicly available RNA-seq data to help nominate sensitive and specific surface proteins and exclude surface proteins expressed in normal tissues.

## Materials and Methods

### RNA-seq Data Processing

RNA-seq data were obtained from previously published datasets (9, 29–31) of NEPC (*n* = 27), metastatic CRPC (*n* = 72), localized prostate cancer (*n* = 68), and benign prostate tissue (*n* = 66). Normal tissue RNA-seq data were obtained from the Genotype-Tissue Expression Project [GTEx (32), *n* = 30]. The GTEx Project was supported by the Common Fund of the Office of the Director of the NIH, and by NCI, NHGRI, NHLBI, NIDA, NIMH, and NINDS. The data used for the analyses described in this article were obtained from: the GTEx Portal on March 1, 2015 and/or dbGaP accession number phs000424.vN.pN on March 1, 2015. Reads were mapped to the human genome reference sequence (hg19/GRC37) using STAR v2.3.0e (33). For each sample, HTSeq (34) and Cufflinks (35) were then used to generate read counts and fragments per kilobase of transcript per million mapped reads (FPKM), respectively. Gene counts and DESeq2 were used to detect differential gene expression between CRPC-NE (neuroendocrine) samples and CRPC-Adeno, prostate cancer, and benign samples (36). Cuffdiff was used to identify differentially expressed transcripts (35).

### IHC

For patient tumor samples, IHC for CEACAM5 was performed using R&D Systems antibody (#487609, mouse monoclonal IgG2a recognizing recombinant human CEACAM5, Lys35-Ala685) at a 1:100 dilution. Formalin-fixed paraffin-embedded (FFPE) tissue sections were deparaffinized and endogenous peroxidase was inactivated. Antigen retrieval was accomplished by the Bond Epitope Retrieval Solution 1 (Leica Biosystems, AR9961) at 99°C to 100°C for 30 minutes. Following retrieval, the sections were incubated sequentially with the primary antibody for 25 minutes, post-primary for 15 minutes and polymer for 25 minutes ending with colorimetric development with diaminobenzidine (DAB) for 10 minutes using the Bond Polymer Refine Detection Kit (Leica Biosystems, DS9800). RHAMM IHC was performed using a rabbit recombinant monoclonal RHAMM antibody (EPR4055; Abcam) on paraffin-embedded tissue sections on a Leica Bond system following the manufacturer's protocol. The sections were pretreated using heat-mediated antigen retrieval with Tris-EDTA buffer (pH = 9, epitope retrieval solution 2) for 20 minutes and incubated with RHAMM antibody (1:100 dilution) for 15 minutes at room temperature. RHAMM was detected using an horseradish peroxidase (HRP)-conjugated compact polymer system and DAB as the chromogen. Each section was counterstained with hematoxylin and mounted with Leica Micromount. RHAMM expression was scored as positive if any staining was present.

All histologic evaluations and quantifications [including hematoxylin and eosin (H&E)-stained and IHC slides] were performed by a board-certified, genitourinary pathologist (B. Robinson) who followed criteria that have been described previously (37). For NEPC organoid xenograft tumors, 4 μm FFPE sections were cut and deparaffinized in xylene solution and gradually rehydrated in ethanol. Then, slides were incubated in preboiled 10 mmol/L sodium citrate buffer (Sigma-Aldrich) for the antigen retrieval and blocked by 3% hydrogen peroxide for 10 minutes at room temperature. VECTASTAIN Elite ABC-HRP Kit (Vector Laboratories) was used to proceed the staining per manufacturer's protocol. Slides were developed using a DAB Substrate Kit (Vector Laboratories #SK-4100) and imaged by a Nikon Eclipse Ti2 microscope. Primary antibodies were used as following: anti-KRT8 (1:50, DSHB #TROMA1), anti-INSM1(1:200, Santa Cruz Biotechnology #SC-377428), anti-ASCL1(1:200, Santa Cruz Biotechnology #SC-374104), anti-SYP(1:500, Sigma-Aldrich #336R-94), anti-CHGA(1:2500, Abcam #ab15160).

### Multiplex CRISPR Knockout of CELSR3 in NEPC Organoids

The sequence of single-guide RNA (sgRNA) targeting cadherin EGF LAG seven-pass G-type receptor 3 (CELSR3) was from Human CRISPR knockout (KO) library (H3) developed by Xiaole Shirley Liu and Myles Brown (Addgene #133914). sgCELSR3-1: ACAGTCGTGCTGCGCGTCA; sgCELSR3-4: GAAAGTAACCTCGGCGAAC; sgCELSR3-5: CGCCACCGATATGCGCCCT. sgRNAs were subcloned into lentiCRISPR v2 plasmid, a gift from Feng Zhang (Addgene plasmid #52961; http://n2t.net/addgene:52961; RRID:Addgene_52961). We then used HEK293FT cells to produce lentiviral supernatants that carried lentiCRISPR v2-sgCELSR3 constructs. To infect WCM154 organoids, we first dissociated organoids to single organoid cells. A total of 1 × 10^6^ cells were plated in a 6-well plate in 1.5 mL HM media. We then added 4 μg/mL of polybrene (Millipore) and 500 μL lentiviral supernatant onto organoid cells. The plate was centrifuged at 600 × *g* and 32°C for 60 minutes, then incubated in 37°C to recover for overnight. Infected organoid cells were selected in 1 μg/mL puromysin for 72–96 hours.

### Short Hairpin RNA Knockdown of CELSR3

We performed lentiviral short hairpin (shRNA) transduction of the NEPC patient-derived organoid (PDO) WMC154 with lentivirus packaged with shRNA targeting CELSR3 (targeting sequence in the 3′ untranslated region) or scrambled sequence as a control as we have described previously (31). Briefly, NEPC PDO WMC154 were dissociated with TrypLE (Gibco) and resuspended in organoid medium (38) containing Polybrene (Millipore) and Y27632 (Selleckchem, S1049). The dissociated organoid cells were combined with viral suspension and centrifugated at 600 × *g*, 32°C for 60 minutes. The organoid/virus mix was then incubated at 37°C overnight. Organoid cells were subsequently collected, resuspended in 120 μL of Matrigel (Corning) and seeded in a 24-well plate. Antibiotic selection was performed using 1 μg/mL puromycin (Thermo Fisher Scientific) for 7 days. LNCaP-N-Myc cells were transduced in 6-well plates in medium containing Polybrene (Millipore). Cells were selected with 2 μg/mL puromycin (Thermo Fisher Scientific) for 7 days and grown without puromycin 1 for 6 weeks.

### Cell Growth Assays

Organoids were dissociated to single cells and 3,000 cells/well were seeded in a collagen-coated 96-well plate. At indicated timepoints, CellTiter-Glo (Promega) was used to examine cell viability. The relative growth was determined by normalized readouts to day 1. Three independent studies were performed and at least four technical duplicates were designed in each independent experiment.

### Organoid Cell Culture and Orthotopic Tumor Cell Injections

Patient-derived NEPC three-dimensional organoids cultures were maintained using a protocol as described previously (38, 39). Eight-week-old male NSG mice (NOD-*scid* IL2Rgamma^null^) were purchased from the Jackson Laboratory and used for all animal experiments in this study. To inject organoids in the mouse prosate, mice were disinfected and a small incision was made on the abdomen, approximately 1 cm from the genital. The seminal vesicle was gently pulled out to expose the anterior prostate. The total volume of 50 μL containing 2 × 10^5^ organoid cells and the equal ratio of matrigel (BD #356231) was slowly injected into the anterior prostate using a 29G insulin syringe. Then the incision was sutured and mice were monitored until they fully recovered from the surgery. After 6 months, when mice reached humane endpoints defined in the protocol, mice were euthanized and necropsy was performed to examine primary tumor and metastasis. The animal study was approved and conducted under Dana-Farber Cancer Institute Institutional Animal Care and Use Committee (IACUC) protocol (18-020).

### Immunoblot

Organoids and tumor lysates were extracted using RIPA buffer (Sigma-Aldrich) in the presence of 1X protease inhibitor (Roche) and phosphatase inhibitor (Thermo Fisher Scientific), and incubated at 4°C for 40 minutes. Lysates were vortexed every 10 minutes to achieve homogeneous digestions. The lysates were spun down, and supernatants containing proteins were collected. Protein concentration was measured using the DC protein assay (Bio-Rad) and 50 μg proteins were subjected to 4%–15% TGX Stain-Free Gels (Bio-Rad) and then transferred onto 0.2-μm nitrocellulose membranes. Membranes were then blocked in 5% nonfat milk. Primary antibodies were used as following: anti-CELSR3 (1:1,000, NOVUS #NBP238975), anti-CHGA (1:1,000, Abcam #ab15160), anti-SYP (1:2,000, Sigma-Aldrich #336R-94), anti-FOXA2 (1:2,000, Abcam #ab108422), anti-SOX2 (1:1,000, Cell Signaling Technology #2748), anti-BRN2 (1:1,000, Cell Signaling Technology #12137), anti-E-cardherin (1:500, Abcam #ab40772), anti-Snail (1:1,000, Cell Signaling Technology #3879), anti-Vinculin (1:5,000, Cell Signaling Technology #13901S). Membranes were then incubated with respective secondary antibodies for 1 hour at room temperature: Anti-Mouse IgG-HRP (1:2,500, Bio-Rad #1706516), Anti-Rabbit IgG-HRP (1:2,500, Bio-Rad #1706515). Membranes were developed using Luminata Western HRP Chemiluminescence Substrates (Millipore) and imaged by clear-blue X-ray films (Thermo Fisher Scientific).

### Immunofluorescent Staining

A total of 4 μm deparaffinized slides were prepared as described above. Alexa Fluor Tyramide SuperBoost Kits (Thermo Fisher Scientific) were used to perform the multiplex staining. Briefly, slides were incubated with a primary antibody for overnight and corresponding secondary antibody for 1 hour. After antibodies incubation, the first AlexaFluor dyes were applied following the manufacturer's protocol. Then, the slides were reboiled in the preboiled sodium citrate solution (10 mmol/L, pH 6.0). Slides were then incubated with another primary antibody for overnight and corresponding secondary antibody for 1 hour. The second AlexaFluor dyes with distinct wavelength were applied. After all primary antibodies conjugations were completed, NucBlue (Thermo Fisher Scientific #R37606) was used to stain nucleus, and then slides were mounted using VECTASHIELD Vibrance Antifade Mounting Medium (Vector Laboratories #H-1700-10). Slides were imaged using a Nikon Eclipse Ti2 microscope. Primary antibodies used for multiplex staining are anti-KRT8 (1:25, DSHB #TROMA1), anti-INSM1(1:50, Santa Cruz Biotechnology #SC-377428), anti-SYP(1:500, Sigma-Aldrich #336R-94).

### Cell Migration Assays

We performed cell migration assays using Corning FluoroBlok cell culture inserts and according to manufacturer's protocol. Briefly, fluorescently-labeled cells were allowed to migrate for up to 48 hours, and the percentage of cells migrated into the plane of view was calculated. Cells that have migrated through the membrane were detected using a bottom-reading fluorescence microscope. Three areas of view per biological replicate (*n* = 2) were counted at 4, 18, 24, and 48 hours postseeding Student *t* tests were performed at each timepoint.

### Laser Capture Microdissection and RNA-seq Analysis

Following region of interest review, carried out by a board-certified pathologist, laser capture microdissection (LCM) was performed on the Molecular Machines & Industries CellCut platform. To ensure maximum RNA was recovered from samples, RNA extraction was carried out using RNEasy FFPE Kit (Qiagen). RNA quality was verified using Agilent Bioanalyzer 2100 (Agilent Technologies). To accommodate the RNA concentration and yields associated with samples microdissected from FFPE tissues, we utilized the Illumina Stranded Total RNA Prep with Ribo-Zero Plus method for library preparation. Paired-end, 150 × 2 cycles sequencing was performed on the NovaSeq 6000 instrument. Quality control of raw sequencing reads was performed using FastQC (Babraham Bioinformatics). Low-quality reads were removed using Trimmomatic (40) with a sliding window size of 4 bp and a quality threshold of 20. The resulting reads were aligned to mm10 using STAR (33). Reads were sorted and indexed using SAMtools (41). Transcript abundance was calculated in FPKM using Cufflinks (35) and in gene counts using HTSeq (34). Differential gene expression was assessed using DESeq2 (36). For variant calling, GATK's best practices pipeline (42, 43) was followed including the alignment method as described previously (44). Reads with less than 30 sequence length were removed before alignment. In brief, reads were aligned to the mm10 reference genome with STAR in paired-end and two-pass mode. PCR duplicates were removed using Picard tools and reads were split into exon segments keeping the grouping information by SplitNCigarReads of GATK (42). Reads were further realigned at known indel positions and base quality score was recalibrated. Haplotype Caller (43) was used for calling variants (both single-nucleotide variants and indels) from each of the murine tumor tissues. Filtered variants (covered by at least 10x depth) were annotated in Annovar (45) using RefSeq gene assembly. All animal studies included in this article have been approved by the Weill Cornell Medicine's IACUC.

### T-cell Killing Assays

TCCSUP (ATCC HTB-5), DU145 (ATCC HTB-81), and TCCSUP CELSR3 KO (generated using CRISPR-Cas9 technology at Synthego using parental TCCSUP), and PM154 at passage 2 post-thaw were transduced with NucLight Red lentivirus (Sartorius #4476) at multiplicity of infection of 3 and incubated for 24 hours. The following day placed in 1 μg/mL puromycin selection for 1 week. NucLight red nuclear expression was confirmed by microscopy. The resulting populations were named TCCSUP NLR, TCCSUP CELSR3 KO NLR, PM154 NLR, and DU145 NLR. TCCSUP KO cells were generated at Synthego using 5,000 cells were seeded in phenol red-free media into 96-well black optical clear collagen-coated plates and incubated overnight. The following day, 25,000 purified pan T cells were added to the cells and placed into an IncuCyte S3 live cell imager. Images were recorded every 6 hours for 7 days and analyzed for the number of red nuclear counts. Percent lysis was calculated by dividing the number of cells in each well by the average number of cells in control untreated wells. Three biological replicates were recorded for each treatment.

### Quantitative Flow Cytometry

Tumor cells lines were removed from flasks using TrpLE Select (Thermo Fisher Scientific #12563029) and washed twice with PBS. A total of 50 μL of a 1:1,000 stock solution of Live/Dead Violet antibody in PBS (Thermo Fisher Scientific #L34963) was added to each sample of cells and incubated for 15 minutes at room temperature in the dark. The cells were washed twice in BD stain buffer (BD Biosciences #554657). A total of 50 μL of a proprietary CELSR3 flow antibody which binds to the GAIN region of CELSR3 was added for a final concentration of 1 μg/mL and incubated for 45 minutes at 4°C in the dark. The cells were washed twice in BD stain buffer. Quantum Simply Cellular anti-mouse IgG beads (Bangs Labs #815) were similarly stained with the proprietary CELSR3 flow antibody. Samples were run on a BD FACSCelesta flow cytometer (BD Biosciences) and mean fluorescence intensities recorded for at least 10,000 events per sample. A CELSR3 standard curve and receptor density for each sample was generated using the Quantum Simply Cellular anti-mouse IgG beads according to the manufacturer's protocol.

### RT-PCR

A total of 1 μL of pooled normal tissue cDNA (Takara #636742 and 636743) was run on a Viia7 RT-PCR instrument (Thermo Fisher Scientific) using three Thermo Taqman primers for CELSR3 (Hs00996904_m1, Hs00609786_g1, Hs00609761_g1). ddCt was calculated for each tissue sample and normalized to 22Rv1 expression.

### Code Availability

The code that was used for the multi-step bioinformatics pipeline to identify NEPC-specific, overexpressed gene transcripts that encode surface proteins is available at: https://github.com/kgayvert/NeuroSurfacePro

### Data Availability Statement

RNA-seq data are from previously published datasets (9, 29–32).

## Results

### Identification of Gene Transcripts Encoding Surface Proteins that are Specifically Upregulated in NEPC

**FIGURE 1:**
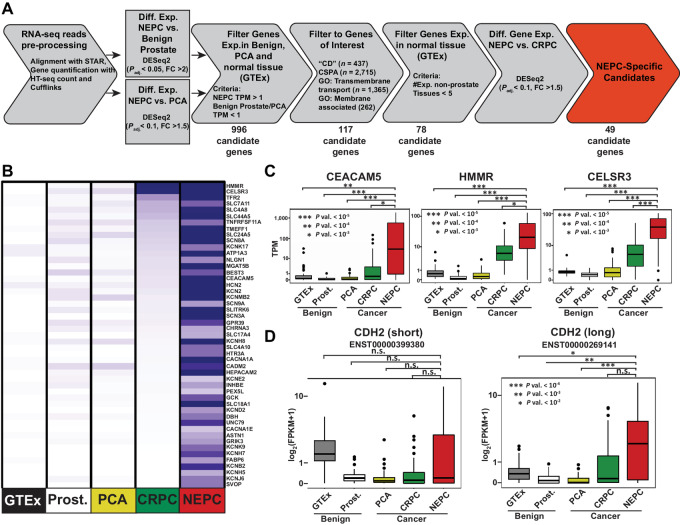
**A,** Schematic of the bioinformatics pipeline that was used to analyze bulk RNA-seq data with the aim of identifying cell surface protein encoding genes specifically enriched in CRPC and NEPC compared with localized prostate cancer (PCA), benign prostate (Prost.) and across the different normal tissues that are represented in the GTEx database. **B,** Heat map summarizing the average expression levels of the indicated genes from RNA-seq data from the normal tissue from the GTEX database, benign prostate, prostate cancer, CRPC, or NEPC tumors from our cohort. Box plot showing distribution of expression of the indicated gene (**C**) or CDH2 transcript isoform (**D**) based on RNA-seq data from the clinical cohort of normal tissue from GTEX, benign prostate, prostate cancer, CRPC, or NEPC. Box represents 25%–75% percentile with median denoted as a line. Whiskers extend ± 1.5× interquartile range. Datapoints that lie outside the whiskers are plotted individually.

On the basis of RNA-seq data of tissues from benign prostate, localized prostate cancer, metastatic CRPC (adenocarcinoma) and NEPC (9, 31), we developed a multi-step bioinformatics pipeline to identify NEPC-specific, overexpressed gene transcripts that encode surface proteins. Using adjusted *P* value and fold-change cut-off criteria to identify transcripts of surface protein genes expressed in NEPC tumors (*n* = 27), but not in critical normal tissues [GTEx Project (32), *n* = 30], benign prostate tissue (*n* = 66), locally advanced prostate cancer (*n* = 68), or CRPC (*n* = 72)). Specifically, RNA-seq data were aligned to the human genome using STAR and was further processed using HTSeq Count and Cufflinks to generate FPKM values for each gene's transcript. We then used DESeq2 to test for differential expression of transcripts comparing the data from NEPC and benign prostate tissue (adjusted *P* value less than 0.05 and a fold-change cutoff of greater than or equal to 2.0). Gene transcripts were filtered out if the transcript per million (TPM) values were greater than 1.0 in the benign prostate tissue dataset or less than in 1.0 in NEPC dataset. We also used DESeq2 to test for differential expression of transcripts comparing the data from NEPC and prostate cancer (adjusted *P* value less than 0.1 and a fold-change cutoff of greater than or equal to 1.5). Gene transcripts were filtered out if the TPM values were greater than 1.0 in the prostate cancer dataset or less than in 1.0 in NEPC dataset. This led to the identification of 996 gene transcripts. We then relied on Human Cell Differentiation Molecules (HCDM), Cell Surface Protein Atlas (CSPA; ref. 46), and Gene Ontology (GO) nomenclature to restrict our analyses to transcripts that encode putative cell surface (genes with a “CD” nomenclature, *n* = 437), mass spectrometric–derived CSPA (*n* = 3152), ion transmembrane transport (GO:0034220, *n* = 246), transmembrane transport (GO:0055085, *n* = 1,119) or membrane-associated (GO:0016020, *n* = 3,160; Supplementary Fig. S1A) proteins. This restricted the list to 117 gene transcripts. As the ultimate goal was to identify targetable surface proteins, we further filtered out gene transcripts if they were expressed in normal tissue. For this, we relied on data from the GTEx (32) dataset, which at the time of these analyses housed expression data from 2,921 samples of 30 different tissue types. For every gene transcript, we first calculated the median TPM for each tissue and eliminated gene transcripts that had a median TPM value that was greater than 3.0 in five or more different tissues. This further reduced the list to 78 gene transcripts. To restrict this list to gene expression that were enriched in NEPC compared with CRPC, we performed DESeq2 (NEPC versus CRPC; adjusted *P* value less than 0.1 and a fold-change cutoff of greater than or equal to 1.5) and identified a subset of these transcripts which were uniquely expressed in NEPC and not in CRPC, which resulted in a final list of 49 gene transcripts (Fig. 1A and B; Supplementary Table S1). This included the identification of known NEPC surface protein CEACAM5 as well as other potentially targetable proteins (e.g., HMMR and CESLR3; Fig. 1C). However, because our approach filtered out any gene whose encoded protein was not assigned any of the cell surface protein nomenclatures in the HCDM, CSPA, or GO databases, genes encoding recently validated NEPC-associated surface proteins such as DLL3 were filtered out.

### Identification of Spliced Transcript Isoforms Encoding Surface Proteins that are Specifically Upregulated in NEPC

Using the same RNA-seq data, we developed an additional bioinformatics approach to identify NEPC-specific, overexpressed gene transcripts that encode surface protein isoforms. For this, we followed the same approach and filtering criteria as described above and used CuffDiff instead of DESeq2. This process started with 66,235 protein coding gene transcript isoforms and after identifying 895 differentially expressed surface-associated gene transcript isoforms in NEPC compared with benign prostate tissue and that are not expressed in normal or prostate cancer tissues, we found that 28 gene transcript isoforms were overexpressed in NEPC compared with CRPC and which included a new transcript isoform encoded by the *CDH2* gene (Fig. 1D). *CDH2* encodes for cadherin 2, type 1, N-cadherin (Neuronal) which is calcium-dependent cell-cell adhesion glycoprotein. N-cadherin has previously been shown to play a causal role in tumor cell invasion, metastasis, and castration resistance (47). This new isoform (ENST00000269141) is different from the most common isoform (ENST00000399380) by the inclusion of additional exon (exon 2) that putatively encodes an additional 57 amino acids that houses a signal peptide and a prodomain (Supplementary Table S1).

### Validation of NEPC-enriched Surface Protein Expression

**FIGURE 2:**
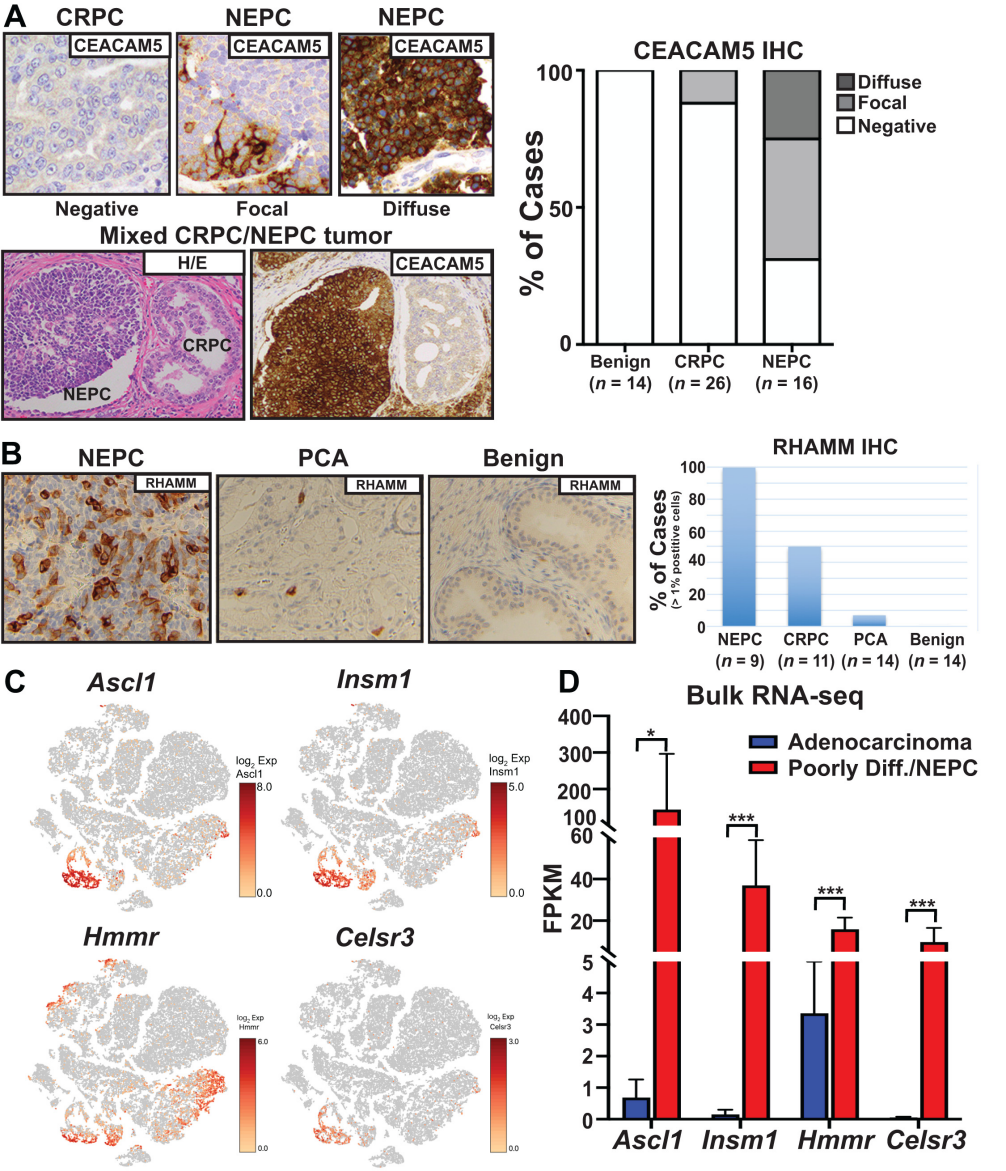
**A** and **B,** Representative IHC staining for CEACAM5 including a mixed foci harboring both NEPC (left) and CRPC (right) that stained positive and negative for CEACAM5, respectively (A) and RHAMM (B) in NEPC and in other tissue samples types from serial sections used in this study (scale bar = 50 μm). **A,** Right: CEACAM5 IHC expression patterns (negative, focal, or diffuse) on TMAs stratified by tissue type (benign prostate, *n* = 14; CRPC, *n* = 26; NEPC, *n* = 16). **C,** Combined t-distributed stochastic neighbor embedding (t-SNE) plot of scRNA-seq data from intact *PRN* (*n* = 3) mice. Shown are the expression of indicated markers or signature in *PR* (*n* = 3) compared to *PRN* GEM (*n* = 3) mice. **D,** RNA-seq FPKM values of the indicated genes which were significantly deregulated in *PRN* adenocarcinoma or poorly differentiated/NEPC tumors. *, *P* < 0.05; **, *P* < 0.01; ***, *P* < 0.0001 (Students *t* test).

CEACAM5 (CD66e) is a carcinoembryonic antigen-related cell adhesion molecule protein that belongs to the immunoglobulin gene superfamily. CEACAM5 is a glycosylated oncofetal antigen, expressed in intestinal epithelial cells of gut allowing interactions with CD8 T cells and, in addition to NEPC (28, 48), has been shown to be overexpressed in gastrointestinal cancers (49). IHC staining of a tissue microarray (TMA) containing triplicate tissue cores from 14 benign prostate tissue samples, 26 CRPC tumors, and 16 NEPC tumors confirmed diffuse or focal strong membrane staining for CEACAM5 in the majority of NEPC tumors and focal membrane staining in only a minority of CRPC tumors (Fig. 2A). None of the benign prostate tissue samples were positive for CEACAM5 expression. We also found that CEACAM5 is overexpressed in patient-derived NEPC organoids that we have previously described [WCM154 (diffuse expression), WCM155 (focal expression), and WCM1078 (focal expression; ref. 38)] (Supplementary Fig. S1B).

We identified the hyaluronan-mediated motility receptor (HMMR) gene *HMMR* (50) as another gene transcript that is expressed specifically in NEPC. *HMMR* encodes for the hyaluronan-mediated motility receptor (RHAMM) and physically interacts with hyaluronan and engages RHAMM signaling. It is a known transcriptional target of TGFβ signaling in sarcoma (51, 52) and has also been linked to YAP1 signaling and breast cancer cell motility (53). RHAMM has also been reported to be induced following *RB1* loss, is an E2F target gene and affords a metastatic potential for prostate cancer cells through the Rho-associated kinase signaling pathway (54). To get an idea of the extent of RHAMM expression in prostate cancer patient tumors, we performed IHC staining of another TMA that contained triplicate tissue cores from 14 benign prostate tissue samples, 14 prostate cancer, 11 CRPC, and nine NEPC tumors. We found that all nine NEPC cases contained tumor cells with membrane positivity for RHAMM (Fig. 2B); however, these cells represented only about 10% of all of the NEPC tumor cells in a given TMA spot (Supplementary Fig. S2). Among the CRPC cases, six of 11 contained scattered RHAMM-positive tumors cells, but the proportion of such RHAMM-positive tumor cells in CRPC was much lower than in NEPC (Fig. 2B). While none of the cells in benign prostatic tissue were RHAMM-positive, there was one exceptional prostate cancer case that had RHAMM-positive tumor cells. Interestingly, the positive prostate cancer tissue was from a patient diagnosed with prostatic adenocarcinoma with a high Gleason score of 9 (5+4), invasion into both the seminal vesicles and periprostatic soft tissue, positive apical and bladder neck margins, and evidence of vascular and perineural invasion. Three years later, the same patient presented with a pelvic mass that was shown to be a poorly differentiated metastatic prostatic adenocarcinoma with neuroendocrine features (Fig. 2A).

CELSR3 was found to be overexpressed in NEPC compared with CRPC, prostate cancer, and benign tissue (Fig. 1C). CELSR3 is part of the larger adhesion-class G protein-coupled receptors. It contains large extracellular domains which are cleaved autoproteolytically at a conserved GPS within the GAIN domain. CELSR3 has been shown to regulate neural precursor cell fate decisions through the Wnt signaling pathway (55) and has been previously identified in small intestinal neuroendocrine tumors (56). CELSR3 gene expression has been linked to a poor prognosis in patients with primary prostate cancer (57) but its expression in advanced CRPC and NEPC has yet to be determined. Unfortunately, there is no commercially available antibody that would allow reliable validation of CELSR3 protein expression in patient samples. To further validate the association between CELSR3 expression and the NEPC phenotype, we queried its expression in a genetically engineered mouse (GEM) model that we reported recently (58). We engineered this GEM with prostate epithelial cell–specific co-loss of Pten and Rb1 and overexpression of the human MYCN gene (or PRN). We reported that PRN mice developed large, invasive primary and metastatic tumors with poorly differentiated/NEPC foci that were AR negative and expressed the neuroendocrine marker INSM1 (31) as early as 8 weeks (58). Single-cell RNA-seq (scRNA-seq) data analyses identified discrete populations of luminal (Ar+/Cd24+) cells that were AR signaling high or *Ezh2*+/AR signaling low suggesting a transition away from an AR-dependent state, consistent with our previous work (31, 59) as well as a subpopulation of NEPC cells (*Ascl1*+ and *Insm1+;*Fig. 2C; ref. 58). On the basis of these data, we also observed that both *Hmmr* and *Celsr3* transcripts were enriched in the NEPC cell population (Fig. 2C). In this model, we also detected *Hmmr* transcripts in the adenocarcinoma cell population. Interestingly, some of the *Hmmr*-positive adenocarcinoma cells were also positive for *Ascl1* and *Insm1* suggesting that this subpopulation of adenocarcinoma cells may have overlapping features or may be in the process of transitioning toward the NEPC phenotype. To determine whether *Hmmr* and *Celsr3* are differentially expressed in the poorly differentiated/NEPC tumor foci compared with the adenocarcinoma tumor foci, we performed bulk RNA-seq on histologically distinct tumor foci (*n* = 6 adenocarcinoma foci and *n* = 6 poorly differentiated/NEPC foci; ref. 58). These poorly differentiated/NEPC tumor foci had transcriptomic NEPC scores (9) that were on par with values obtained from a cohort of patient NEPC tumors (58). On the basis of this dataset, we found that both *Hmmr* and *Celsr3* were expressed at significantly higher levels in the differentiated/NEPC tumor foci compared with the adenocarcinoma tumor foci (Fig. 2D).

### CELSR3 Knockdown Results in Reduced NEPC Tumor Cell Proliferation and Migration *In Vitro*

**FIGURE 3:**
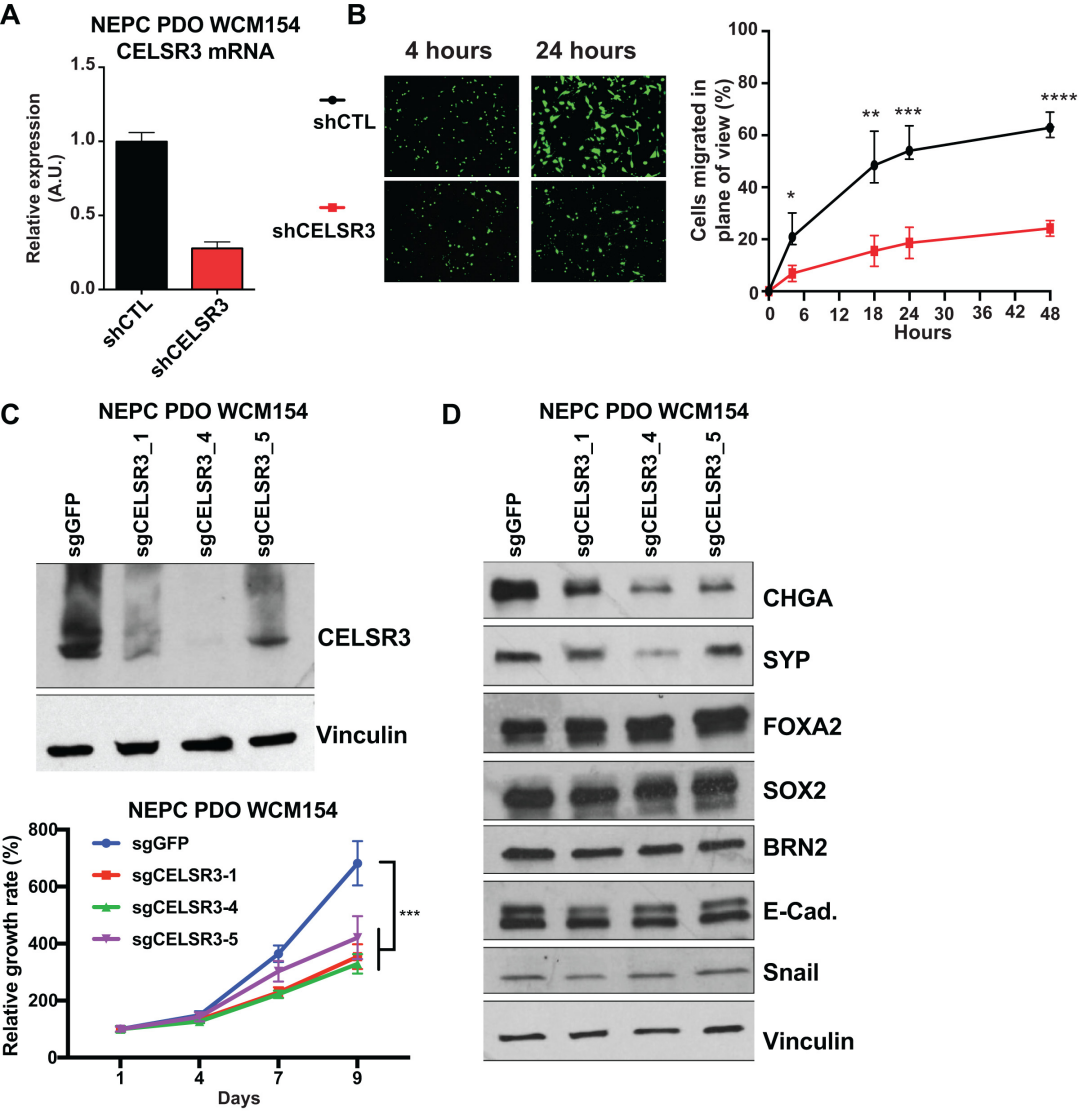
**A,** Quantitative RT-PCR of mRNA expression of CELSR3 following stable expression of hairpin molecules targeting CELSR3 (shCELSR3) or control non-targeting hairpin molecules (shCTL) in the NEPC PDO WCM154. **B,** Left: Representative images of migrated NEPC PDO WMC154 cells 4 or 48 hours postseeding using Corning FluoroBlok cell culture inserts. Right: Average and SD of the cell counts from the cell migration assays at 4, 18, 24, and 48 hours postseeding. The data are a summary from three plane views per biological replicate were counted at 4, 18, 24, and 48 hours postseeding. *, *P* < 10^4^; **, *P* < 10^6^; ***, *P* < 10^7^; ****, *P* < 10^8^ (Student *t* tests). **C,** Protein levels and proliferation curves of NEPC PDO WCM154 organoids following CRISPR/Cas9 KO using guide RNAs targeting *CELSR3* (sgCELSR3) or GFP (sgGFP) as a control. **D,** Western blot analysis results showing the expression of the indicated NEPC markers following CELSR3 KO as described in C.

CELSR3 has previously been implicated in axonogenesis, neuron migration, and cell-cell adhesion, all of which are involved in the process of perineural invasion in oral squamous cell carcinoma (60) and neuroblast migration in postnatal brain (61). To determine whether CELSR3 plays a functional role in NEPC cell growth or migration, we performed genetic knockdown of CELSR3 either targeting its mRNA using shRNA (Fig. 3A) or through gene editing using CRISPR/Cas9 technology using three independent sgRNA targeting CELSR3 (sgCELSR3_1, sgCELSR3_4 and sgCELSR3_5) in WCM154 NEPC PDO (Fig. 3C). Control shRNA and sgGFP engineered cells were used as negative controls. From this, we found that reducing CELSR3 resulted in reduced cell migration (*P* < 1.0 × 10^−11^, Student *t* test) and cell proliferation (*P* < 0.0001; Fig. 3B and C). We also found that CELSR3 KO resulted in a reduction of the NEPC markers CHGA and SYP but did not impact the levels of NEPC-related transcription factors, such as FOXA2 (62), BRN2 (63) or SOX2 (64) or the epithelial-to-mesenchymal transition (EMT) markers E-cadherin or Snail (Fig. 3D).

### CELSR3 KO Suppresses the NEPC Phenotype *In Vivo*

**FIGURE 4:**
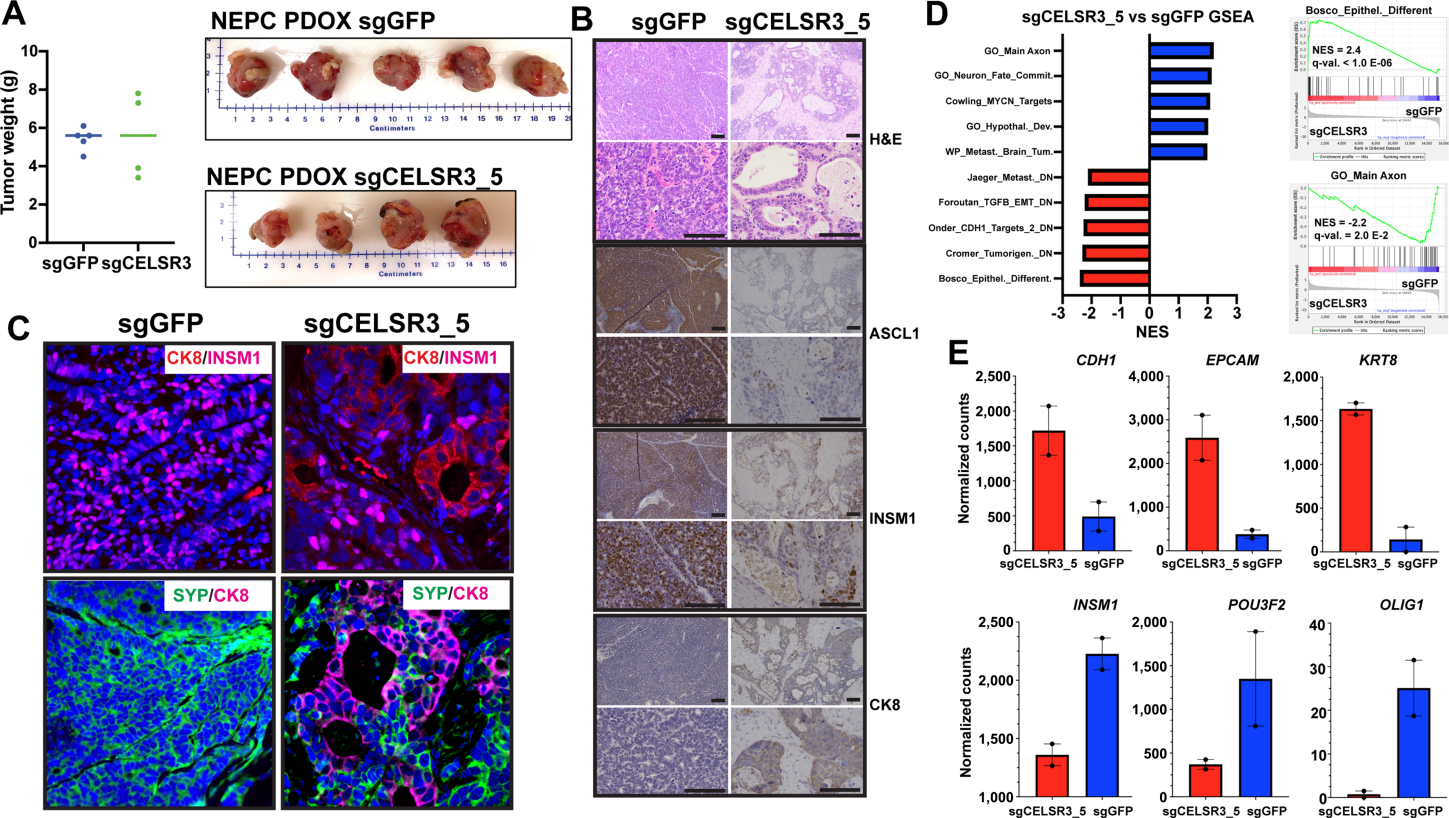
**A,** Final tumor weights and images of WCM154-sgGFP and WCM154-sgCELSR3 PDOX models. **B,** H&E and IHC staining of indicated markers (scale bar: 50 μm) from serial sections. **C,** Multiplex immunofluorescence of lineage markers of sgGFP and sgCELSR3 PDOXs. **D,** Top gene sets enriched from the GSEAs of the RNA-seq data analyses from the sgCELSR3 PDOX laser capture cells (red) compared with sgGFP captured cells (blue). Right: GSEA enrichment plots of Bosco epithelial differentiation gene set or the GO_Main_Axon gene set enriched sgCELSR3 PDOX laser capture cells or the sgGFP captured cells, respectively. **E,** Normalized RNA-seq counts obtained for the indicated gene in sgCELSR3 PDOX laser capture cells (red) or the sgGFP captured cells (blue).

Because CELSR3 inhibition resulted in reduced cell proliferation, migration, and expression of NEPC markers *in vitro*, we performed orthotopic injections of WCM154 sgGFP or WCM154 CELSR3 WCM154 NEPC PDO into the anterior lobe of the prostate in 8-week-old immunocompromised mice (*n* = 5 mice per WCM154 sgCELSR3 or WCM154 sgGFP clone). Mice were sacrificed at 6 months postinjection and the tumors were harvested. Upon harvesting, tumors were measured and metastases were counted in each animal. We observed significantly reduced levels of CELSR3 protein in the majority of tumors irrespective of WCM154 sgCELSR3 clones (Supplementary Fig. S3A). Although, we found that the sgCELSR3 decreased organoid growth *in vitro* comparing with sgGFP control (Fig. 3C), there was no significant impact on tumor growth or metastatic potential upon CELSR3 KO (Fig. 4A; Supplementary Fig. S3B). Interestingly, we did observe that WCM154 sgCELSR3 PDO xenograft (PDOX) tumors displayed significantly different tumor histology compared with the sgGFP tumors (Fig. 4B). On the basis of H&E staining, we found that all of the WCM154 sgGFP tumors showed pure small cell carcinoma NEPC morphology, as expected. However, we found that WCM154 sgCELSR3 tumors showed a variety of histologies comprised of regions of small cell/NEPC cells and regions of adenocarcinoma that harbored luminal/gland-like structures (Fig. 4B). We further characterized these different regions and observed a significant reduction of NEPC marker protein expression including ASLC1, INSM1, SYP, and CHGA and an increased level of the luminal epithelial marker cytokeratin-8 (CK8) in the sgCELSR3 adenocarcinoma regions compared with the NEPC regions or sgGFP tumors (Fig. 4B; Supplementary Fig. S3C). This pattern was observed in mice harboring WCM154 sgCELSR3 tumors with different guides (Supplementary Fig. S3D). To further confirm whether the NEPC and adenocarcinoma markers are expressed in distinct cells, we performed immunofluorescence to costain for the neuroendocrine markers SYP and INSM1 along with the epithelial marker CK8 (Fig. 4C). Our data show that the CK8-positive cells did not express SYP or INSM1. Importantly, we did not observe CK8-positive cells in any of the sgGFP tumors. To further characterize the molecular program associated with the CELSR3 KO and appearance of the CK8-positive, adenocarcinoma-like tumor foci, we performed LCM to specifically isolate adenocarcinoma-like cells in sgCELSR3 tumors or NEPC cells in control (sgGFP) tumors and performed whole transcriptome sequencing (RNA-seq). For this, we captured a total of two separate regions of sgCELRS3, adenocarcinoma-like cells as well as two separate regions of sgGFP NEPC cells from FFPE tissue sections. RNA-seq data from each batch of tumor foci were pooled for further analyses. On the basis of gene set enrichment analyses (GSEA), we found that the sgCELSR3 adenocarcinoma-like tumor cells were enriched for expression of genes associated with epithelial differentiation and genes downregulated in the EMT upon TGFβ stimulation (Fig. 4D). We also found that the sgCELSR3 adenocarcinoma-like tumor foci were depleted of expression of neural lineage–related genes. In agreement with these data and the IHC data, we also found that epithelial markers *KRT8*, *EPCAM,* and *CDH1* gene expression levels were upregulated while NEPC-associated genes *INSM1*, *POU3F2* (encodes BRN2) and the neuronal gene *OLIG2* were downregulated in sgCELSR3 adenocarcinoma-like cells compared with sgGFP NEPC cells (Fig. 4E). Although these data suggest that CELSR3 KO results in the upregulation of luminal epithelial molecular program and that the CK8-positive sgCELSR3 cells may have evolved from the SYP/INSM1 cells, more work is needed to understand mechanistically the causative role of CELSR3 for the development or maintenance of the NEPC phenotype.

### CELSR3 is a Target for T-cell Redirection Therapeutics

**FIGURE 5:**
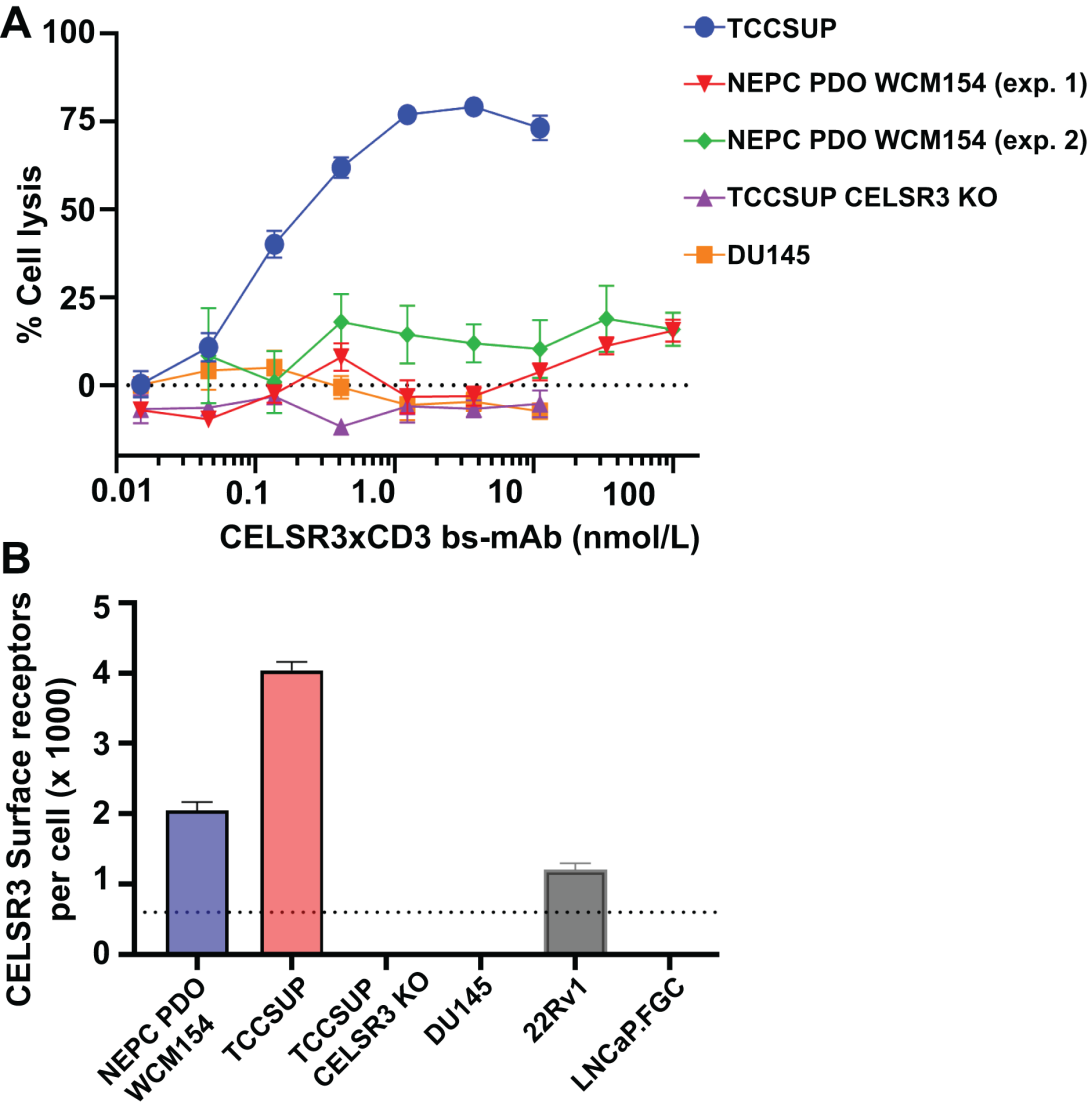
CELSR3 is a target for T-cell redirection therapeutics. **A,** CELSR3xCD3 bs-mAb directs T-cell mediated cytotoxicity in CELSR3(+) TCCSUP NLR and PM154 NLR but not CELSR3(−) DU145 NLR, TCCSUP CELSR3 KO cell lines. 5:1 (exp. 1) or 10:1 (exp. 2) pan T cells to tumor cells were added to each well along with CELSR3xCD3 bs-mAb and imaged using the IncuCyte S3 platform. Maximum cell lysis occurred by day 7 timepoint. At least three biological replicates were analyzed from one independent experiment. **B,** Cell surface expression of CELSR3 on tumor cell lines determined by quantitative flow cytometry and calculated from at least 10,000 events per sample. Dotted line denotes assay lower limit of detection.

*CELSR3* expression is enriched in NEPC, but not in normal tissues (Fig. 1B and C; Supplementary Fig. S1B, GTEx). Therefore, we sought to determine whether CELSR3 was a potential target for T-cell redirection therapeutics. An antibody generation campaign was initiated to identify binders to the GAIN region of the CELSR3 protein. Hits were triaged in biophysical and cellular assays and several potent binders identified and expressed as bispecific mAbs (bs-mAb) designed to simultaneously bind to CELSR3 on a tumor cell and the CD3 receptor present on human T cells. Addition of a CELSR3xCD3 bs-mAb and purified human pan T cells to the CELSR3+ TCCSUP and PM154 cell lines led to 83% and 19% maximum cell lysis by 7 days posttreatment (EC_50_ = 95 pmol/L and 190 pmol/L, respectively). In contrast, treatment with CELSR3xCD3 bs-mAb and T cells did not lead to lysis in the CELSR3(−) DU145 cell line (Fig. 5A). We performed the experiment two times using the NEPC organoid line PM154, once with five T cells per PM154 cell from one validated potent T-cell donor and the second using 10 T cells per PM154 cell and from a different validated potent T-cell donor. CELSR3 surface expression in TCCSUP and PM154 quantified by flow cytometry was approximately 4,000 and 2,000 receptors per cell, respectively. These data indicate that a relatively low number of receptors is sufficient to induce T cell–mediated cytolysis but that a threshold above 2,000 is necessary to mediate more potent cell killing (Fig. 5B). The absence of off-target activity against CELSR3(−) DU145, combined with the low level of expression in normal adult tissues in GTEx and confirmed by targeted RT-PCR with three different Taqman probes (Fig. 5C) suggests that a CELSR3 T-cell redirection therapeutic could be effective in the clinic for patients possessing CELSR3(+) tumors.

## Discussion

There is a need to develop new refined therapeutic strategies for tumor subgroups that are lethal and for which there are no effective treatment options. NEPC is an aggressive subtype of prostate cancer that currently lacks effective therapy. To address this unmet clinical need, we took a bioinformatics approach to identify novel therapeutic targets. Cell surface proteins have the potential to provide an exciting landscape of new tumor subgroup-specific therapeutic targets. They may be targeted using rapidly evolving antibody-mediated therapies such as radionucleotides, ADCs, CAR-T, bispecific antibodies, or other approaches. Cell surface proteins also have potential as molecular imaging biomarkers. For prostate cancer, most of the advance has been made in therapeutics targeting the cell surface protein PSMA (21–26), a marker that is expressed in the vast majority of prostate adenocarcinomas. PSMA is detectable by PSMA PET imaging and is also targeted using the approved radionuclide therapy Lu-PSMA-617, and other approaches in development (e.g., actinium-PSMA, T-cell engagers, CAR-T). Targeting other surface proteins, such as prostate stem cell antigen (refs. 65, 66; NCT03927573, NCT03873805) and TROP2 (ref. 67; NCT04152499) are also in development in prostate cancer. For NEPC, we previously reported the overexpression of DLL3, an inhibitory notch ligand, in the vast majority of NEPC (27). DLL3 is also overexpressed in other poorly differentiated neuroendocrine cancers including SCLC and small cell bladder cancer (68, 69) and not expressed in benign tissues, and there are current clinical trials of DLL3-targeted T-cell engager therapies for patients with NEPC, SCLC, and other DLL3-expressing neuroendocrine carcinomas (NCT04429087, NCT04702737, NCT04471727). Similar to PSMA, it may also be feasible to image DLL3 via PET imaging (70). CEACAM5 is another cell surface target previously reported in NEPC. Therefore, although there has been progress in identifying cell surface proteins in NEPC (48, 71), more work is needed to identify the full spectrum of potentially targetable NEPC-specific surface proteins. Other avenues that may provide additional and pertinent data to nominate NEPC-specific neopeptides could be through direct proteomics (72) or a combination of transcriptomic and proteomic (73) approaches.

Here, we describe the identification of surface protein-encoding genes whose expression is enriched in NEPC compared with CRPC, locally advanced prostate cancer, benign prostate tissue or tissue from other organs. For this, we performed a bioinformatics analysis of RNA-seq data obtained from a large cohort of well-characterized tissue samples of benign prostate, localized prostate adenocarcinoma, CRPC, or NEPC tumors (9, 31). Our analyses also included screening out genes that are expressed in normal tissue beyond prostate using transcriptomic data from GTEx (32). Limitations of this approach are that it could filter out relevant targets expressed in some normal tissues at lower levels but may still be attractive therapeutic targets or not consider targets that are not annotated as a cell surface protein in the HCDM, CSPA, or GO databases. Also there are other methods that could nominate therapeutic targets including outlier analysis that may complement this approach.

On the basis of our pipeline, we identified gene transcripts (e.g., *CEACAM5, HMMR,* and *CELSR3*) and transcript isoforms (new isoform of *CDH2*) that encode surface proteins and whose expression is enriched in NEPC. CEACAM5 has previously been linked to NEPC and has been shown to be an effective target for killing NEPC cells (28, 48) as well as other cancer types such as colorectal and pancreatic cancer (74, 75). We further validated CEACAM5 expression in NEPC and found that while some of the CEACAM5-positive tumors displayed strong diffuse expression of CEACAM5 in all tumors cells, a significant number of NEPC tumors and some CRPC tumors showed a focal pattern of CEACAM5 positivity. We also found that while *HMMR* (encodes RHAMM) gene expression was robustly increased in NEPC tumors, only a subset of the tumor cells were positive for RHAMM protein expression. It is unclear how effective therapies targeting CEACAM5 or RHAMM would be for patients harboring tumors with focal expression, and may also depend on the type of targeting approach developed (e.g., ADC, T-cell engager, or other).

We also discovered *CELSR3* to be overexpressed in NEPC compared with CRPC, prostate cancer, and benign tissue. Although, the lack of an available antibody did not allow for the examination of CELSR3 protein expression in patient tumors, we showed that *CELSR3* knockdown results in reduced NEPC tumor cell proliferation and migration *in vitro*. This is consistent with previous findings that have implicated CELSR3 in axonogenesis, neuron migration, and cell-cell adhesion in oral squamous cell carcinoma cells (60) as well as in neuroblast migration in the postnatal brain (61). *In vivo*, although reduction of CELSR3 did not impact NEPC tumor growth rate or metastatic potential, we did find that reduction of CELSR3 resulted in a diminution of NEPC markers (e.g., SYP, ASCL1, INSM1, SYP, and CHGA) *in vitro* and *in vivo*. Consistent with this, we also found that tumors with reduced levels of CELSR3 displayed a variety of tumor histologies including regions of small cell/NEPC cells and regions that harbored luminal/gland-like structures. These luminal-like regions harbored cells highly positive for the luminal cell marker CK8 and negative for SYP and INSM1. Moreover, LCM-derived RNA-seq analysis confirmed that these KRT-8-high, SYP-low adenocarcinoma cells expressed gene signatures associated with epithelial differentiation and were depleted of neural lineage–related gene expression. Although these data suggest that CELSR3 may play a causal role in the development and/or maintenance of the NEPC phenotype, more mechanistic studies are needed to determine precisely the signaling pathway that is involved in the CELSR3-mediated process. Previously, CELSR3 has been implicated in the regulation of neural precursor cell fate decisions and through a JNK-dependent, noncanonical Wnt signaling pathway (55). Whether the same CELSR3/JNK/Wnt signaling pathway is at play in NEPC has yet to be determined, and further work may identify a targetable essential protein that mediates the role of CESLR3 in NEPC biology.

We have provided initial data that suggests that CELSR3 is a potential target for T-cell redirection therapeutics. Following an antibody generation campaign that identified binders to the GAIN region of the CELSR3 protein, we generated a tool CELSR3xCD3 bs-mAb that demonstrated potent cytolysis of the CELSR3+ TCCSUP cell line. We observed 19% maximum killing against PM154, likely due to a 50% lower CELSR3 receptor density compared with TCCSUP. The relationship between receptor density and induction of T-cell effector function has been studied extensively (76). We hypothesize that the lower killing reflects a critical floor in receptor expression below which there is a significant reduction in the effectiveness of our CELSR3xCD3 bs-mAb. Receptor expression of prostate tumor specific antigens has been shown to increase in more physiologically-relevant contexts, such as spheroids and xenograft models. Future studies could evaluate whether cytolysis is improved in these models where CELSR3 expression is increased to more disease-relevant levels. In addition, an optimized version of our tool CELSR3xCD3 bs-mAb would likely increase cytolysis by more effectively binding to the CELSR3 protein and activating T-cell effector function even at PM154 CELSR3 surface expression levels. Further work is now needed to fully evaluate the utility of targeting CELSR3 with T-cell redirection or other similar therapeutics as a potential new strategy for patients with NEPC.

## Supplementary Material

Supplementary Data Legends Supplementary Table 1 and Supplmentary Figures 1-3 Legends

Supplementary Table 1 Supplementary Table 1. DESeq2 results of all the pairwise comparisons of the tissue groups outlined in Figure 1.

Supplementary Figure 1 Supplementary Figure 1. A. Log fold change ratio (y axis) and mean average expression (x axis) for each gene expression measurements from the bulk RNA-seq data from NEPC tumors compared to locally advanced prostate cancer (PCa) in two samples across all genes (left) or stratified by the indicated annotations for the encoded proteins (right). B. CELSR3 mRNA expression is limited in normal human tissues. mRNA expression measured using RT-PCR and analyzed using the DDCt method with values normalized to 22Rv1 expression (=1) for all tissues. C. Representative IHC staining for CEACAM5 in three different NEPC patient-derived organoids (10x (upper row) and 40x (lower row) magnification).

Supplementary Figure 2 Supplementary Figure 2. Percentage of RHAMM positive cells that were detected using IHC in the indicate tumor type or benign prostate tissue.

Supplementary Figure 3 Supplementary Figure 3. A. Western blot analysis results showing the expression of CELSR3 following CELSR3 or GFP knock-out in the indicated NEPC PDO xenograft tumors for each of the mice (m) that were part of the experiment as described for Figure 3. B. Graphic representation of the percentage of orthotopic NEPC PDO xenograft clones that were engineered with either sgCELSR3 or sgGFP CRISPR-Cas9 that formed macro-metastatic lesions at the indicated region of the mice. C. and D. H&E and IHC staining of the indicated markers.
