# Supplementary material for: The Identification of CELSR3 and Other Potential Cell Surface Targets in Neuroendocrine Prostate Cancer
Source: Cancer Res Commun. 2023 Aug 3;3(8):1447–59. doi: 10.1158/2767-9764.CRC-22-0491 (PMC10401480; doi:10.1158/2767-9764.CRC-22-0491)
Supplement: Supplementary Data Legends — Supplementary Table 1 and Supplmentary Figures 1-3 Legends [file crc-22-0491-s01.docx]

**Supplementary** **Table 1:**

DESeq2 results of all the pairwise comparisons of the tissue groups outlined in Figure 1. Shown are the top expressed genes or transcripts (NEPC versus CRPC or benign) along with, median expression level in the indicated group, log_2_(Fold change) values and associated p values.

**Supplementary Figure Legends:**

**Supplementary** **Figure 1. A.** Log fold change ratio (y axis) and mean average expression (x axis) for each gene expression measurements from the bulk RNA-seq data from NEPC tumors compared to locally advanced prostate cancer (PCa) in two samples across all genes (left) or stratified by the indicated annotations for the encoded proteins (right). **B.** CELSR3 mRNA expression is limited in normal human tissues. mRNA expression measured using RT-PCR and analyzed using the DDCt method with values normalized to 22Rv1 expression (=1) for all tissues. **C.** Representative IHC staining for CEACAM5 in three different NEPC patient-derived organoids (10x (upper row) and 40x (lower row) magnification).

**Supplementary** **Figure 2.** Percentage of RHAMM positive cells that were detected using IHC in the indicate tumor type or benign prostate tissue.

**Supplementary** **Figure 3. A.** Western blot analysis results showing the expression of CELSR3 following CELSR3 or GFP knock-out in the indicated NEPC PDO xenograft tumors for each of the mice (m) that were part of the experiment as described for Figure 3. **B.** Graphic representation of the percentage of orthotopic NEPC PDO xenograft clones that were engineered with either sgCELSR3 or sgGFP CRISPR-Cas9 that formed macro-metastatic lesions at the indicated region of the mice. **C.** and **D.** H&E and IHC staining of the indicated markers (Scale bar: 50 μm).
